# TREM2 in Macrophages Promotes Renal Fibrosis via Activation of β‐Catenin Signalling Pathway in Obstructive Nephropathy

**DOI:** 10.1111/cpr.70192

**Published:** 2026-03-07

**Authors:** Jia Wei, Zixia Li, Gengyu Du, Ting Chen, Min Yang, Zhen Yuan, Yidan Zheng, Xiang Yan

**Affiliations:** ^1^ Department of Urology, Children's Hospital Zhejiang University School of Medicine, National Clinical Research Center for Child Health Hangzhou China; ^2^ Department of Pathology, Children's Hospital Zhejiang University School of Medicine, National Clinical Research Center for Child Health Hangzhou China; ^3^ Department of Cardiology The Second Affiliated Hospital, Zhejiang University School of Medicine Hangzhou China; ^4^ Department of Cardiovascular Surgery Union Hospital, Tongji Medical College, Huazhong University of Science and Technology Wuhan China

**Keywords:** macrophage, obstructive nephropathy, renal fibrosis, TREM2

## Abstract

Obstructive nephropathy leads to renal fibrosis, and Triggering Receptor Expressed on Myeloid Cells 2 (TREM2) drives this macrophage‐mediated process, but its mechanism remains unclear. This study investigated TREM2's role in macrophage polarisation and renal fibrosis progression. In human fibrotic kidneys, TREM2 expression was significantly elevated and co‐localised with macrophages. Unilateral ureteral obstruction (UUO) modelling in mice recapitulated this upregulation, accompanied by renal fibrosis, M2 macrophage polarisation and glomerular filtration rate (GFR) reduction. *Trem2* deficiency (*Trem2* ^*−/−*^) significantly attenuated these pathological changes in UUO mice, preserving GFR. Separately, TREM2 inhibitory peptide sequence IA9 administration reduced renal fibrosis and M2 polarisation in UUO mice. In bone marrow‐derived macrophages (BMDMs), *Trem2* deficiency suppressed IL‐4/IL‐13‐induced M2 polarisation, migration and β‐catenin expression. Critically, lithium chloride (LiCl)‐mediated β‐catenin stabilisation rescued these impairments in *Trem2* ^*−/−*^ BMDMs. In conclusion, TREM2 promotes renal fibrosis by activating β‐catenin signalling to drive profibrotic M2 macrophage responses, establishing TREM2 blockade as a therapeutic strategy for obstructive nephropathy.

## Graphical abstract

Triggering Receptor Expressed on Myeloid Cells 2 (TREM2) promotes obstructive nephropathy progression by activating the β‐catenin signalling pathway, driving M2 macrophage polarisation, migration and fibrogenic gene expression. targeted inhibition of TREM2 thus represents a promising novel strategy against renal fibrosis.

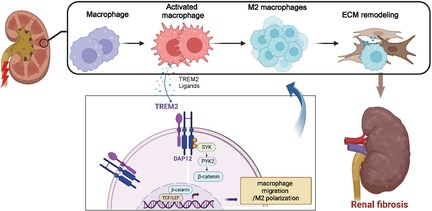

## 1 Introduction

Obstructive nephropathy is a group of diseases caused by congenital or acquired obstruction of the urinary tract, leading to impaired urine drainage, renal fibrosis and persistent renal injury [1]. If not treated promptly, it may progress to chronic kidney disease (CKD) and ultimately end‐stage renal disease (ESRD) [2], significantly impacting patients' quality of life and expectancy along with imposing a substantial economic burden [3, 4]. Current research demonstrated that patients continue to exhibit a progressive decline in renal function even after relief of obstruction [2]. Therefore, elucidating the pathogenesis underlying the progression from obstructive nephropathy to CKD is imperative to identify novel therapeutic strategies.

CKD is pathologically characterised by renal interstitial inflammation and fibrosis, manifested as the pathological proliferation and deposition of fibroblasts and extracellular matrix (ECM) [2]. Studies have established that macrophages play pivotal regulatory roles in renal fibrosis [5, 6]. Notably, M2 macrophages exhibit potent profibrotic functions. These cells secrete critical cytokines such as interleukin‐10 (IL‐10) and transforming growth factor‐β (TGF‐β), stimulate fibroblast proliferation, upregulate collagen synthesis‐related genes in fibroblasts and ultimately promote massive collagen production, thereby exacerbating renal fibrosis.

Triggering Receptor Expressed on Myeloid cells‐2 (TREM2), a member of the TREM family, is predominantly expressed in myeloid‐derived cells including macrophages, dendritic cells and microglia [7]. TREM2 exerts immunomodulatory functions by suppressing pro‐inflammatory cytokine expression and secretion while promoting tissue repair and collagen synthesis. Accumulating evidence has implicated macrophage TREM2 in fibrotic processes. However, its mechanism and fibrogenic impacts appear organ‐specific [7, 8, 9, 10]. TREM2 deficiency was shown to inhibit M2 macrophage polarization, thereby attenuating pulmonary fibrosis [11], whereas TREM2^+^ macrophages suppress hepatic fibrosis progression in non‐alcoholic steatohepatitis [10, 12]. Although recent studies have reported the involvement of macrophage‐derived TREM2 in renal fibrosis, their conclusions remain conflicting [13, 14].

Glomerular filtration rate (GFR) serves as the gold standard for assessing renal function and is critical for the diagnosis and staging of kidney disease. In clinical practice, GFR is frequently estimated (eGFR) using endogenous filtration markers such as serum creatinine (Scr) or cystatin C [15, 16]. However, this approach has significant limitations: Scr concentrations and urine output can be influenced by extra‐renal factors (e.g., hypovolemia, dehydration, protein catabolism), potentially leading to misdiagnosis or delayed detection of acute kidney injury (AKI) [17]. To overcome these constraints, we employed transcutaneous GFR monitoring (tGFR) in this study. This technique non‐invasively measures the elimination kinetics of the exogenous renal marker fluorescein isothiocyanate (FITC)‐sinistrin via an optical device, thereby obviating the need for plasma or urine collection in mice. tGFR provides enhanced precision and reliability compared to conventional methods [18] and has been validated across diverse murine models [19].

Therefore, this study aims to address the functions of TREM2 in renal fibrosis and elucidate its underlying molecular mechanisms, whereas employing tGFR monitoring to assess renal functional changes in mice with obstructive nephropathy.

## 2 Results

### 2.1 TREM2 Expression Was Significantly Elevated and Predominantly Localised to Macrophages in Kidneys From Patients With Obstructive Nephropathy

**FIGURE 1:**
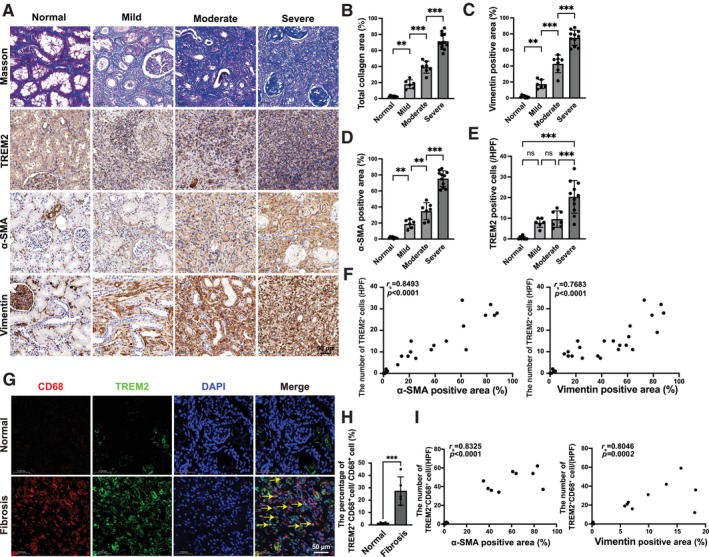
TREM2 was elevated in kidneys from patients with obstructive nephropathy. (A) Masson trichrome staining and immunohistochemistry (IHC) staining for α‐SMA, Vimentin, and TREM2 in kidney tissues with varying degrees of fibrosis. Typical images and quantification are included. Bars = 50 μm. (B–D) With the progression of fibrosis, collagen deposition (B), Vimentin (C), and α‐SMA (D) positive areas gradually increased (*n* = 6–11). (E) Semiquantitative population of TREM2 positive cells in kidneys with varying degrees of fibrosis. The number of TREM2 positive cells was increased in fibrotic tissues (*n* = 6–11). (F) Spearman correlation analysis showed that the number of TREM2^+^ cells was significantly positively correlated with the expression of α‐SMA (*r*_*s*_ = 0.8493, *p* < 0.0001) (*n* = 21) and Vimentin (*r*_*s*_ = 0.7683, *p* < 0.0001) (*n* = 28). (G, H) Representative images of dual immunofluorescence (IF) using CD68 (red), TREM2 (green), and DAPI (blue) with normal kidney and fibrotic kidney. Yellow arrows indicated double positive cells. Randomly selected 10 fields of each patient's kidney under 400× magnification were scored for quantification (*n* = 5). Bars = 50 μm. (I) The number of TREM2^+^CD68^+^ cells was significantly positively correlated with the α‐SMA positive areas (*r*_*s*_ = 0.8325, *p* < 0.0001) and Vimentin positive area (*r*_*s*_ = 0.8046, *p* = 0.0002) (*n* = 15). Data are expressed as the mean ± SD. *p < 0.05; **p < 0.01; ***p < 0.001. HPF, high‐power field.

To investigate the role of TREM2 in renal fibrosis associated with obstructive nephropathy, 36 patients were enrolled, including 24 in the experimental group (renal biopsy or nephrectomy specimens from obstructive nephropathy patients) and 12 controls (histologically normal kidney tissues from peritumoral kidney tissues that had not undergone chemotherapy). Based on fibrotic area occupying < 25%, 25%–50%, or > 50% of cortical area, obstructive nephropathy objects were stratified into mild, moderate and severe fibrosis groups [20, 21]. Immunohistochemistry (IHC) analysis revealed significantly increased expression of α‐SMA, Vimentin and TREM2 with progressive fibrosis severity (Figure 1A–E). Spearman correlation demonstrated strong positive associations between the number of TREM2^+^ cells and both α‐SMA‐positive area (*r*_*s*_ = 0.8493, *p* < 0.0001) and Vimentin‐positive area (*r*_*s*_ = 0.7683, *p* < 0.0001) (Figure 1F).

To evaluate TREM2 expression and localization in kidneys from obstructive nephropathy patients, we performed immunofluorescence (IF) staining. TREM2 staining was localised to the same regions as CD68 staining, demonstrating that TREM2 was predominantly co‐localised in CD68^+^ macrophages (Figure 1G). This suggested extensive infiltration of TREM2‐expressing macrophages within fibrotic renal parenchyma. Quantitative analysis showed markedly elevated proportions of TREM2^+^CD68^+^ cells relative to total CD68^+^ macrophages in fibrotic kidneys (Figure 1H). Spearman correlation confirmed a strong positive association between the number of TREM2^+^CD68^+^ cells and both α‐SMA positive area (*r*_s_ = 0.8325, *p* < 0.0001) and Vimentin‐positive area (*r*_*s*_ = 0.8046, *p* = 0.0002) (Figure 1I).

### 2.2 TREM2 Expression Was Significantly Increased in the Kidneys of UUO Mice and Co‐Localised in Macrophages

**FIGURE 2:**
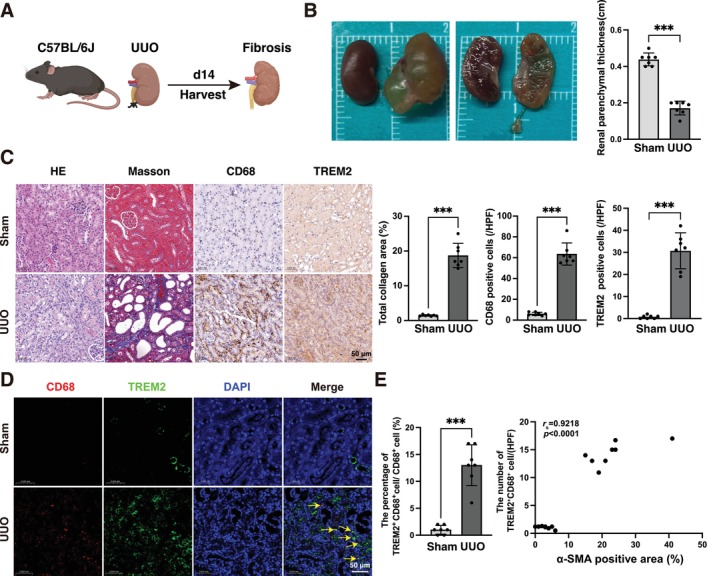
TREM2 expression was markedly increased in the kidneys of UUO mice and co‐localised in macrophages. (A) A schematic diagram showing the procedure of UUO for experimental mice. (B) Gross anatomy of kidney in Sham and UUO. The renal parenchyma in the 14d after UUO group was significantly thinner compared to the sham‐operated group (*n* = 7). (C) Representative H&E, Masson staining and IHC staining images of CD68 and TREM2 in Sham and UUO renal tissues (*n* = 7). Typical images and quantification are included. Bars = 50 μm. (D) Representative images of Sham and UUO dual IF using CD68 (red), TREM2 (green) and DAPI (blue) in Sham and UUO renal tissues. Bars = 50 μm. (E) The number of TREM2^+^CD68^+^ cells was significantly positively correlated with the α‐SMA positive areas (*r*_*s*_ = 0.9218, *p* < 0.0001) (*n* = 16). Data are expressed as the mean ± SD. *p < 0.05; **p < 0.01; ***p < 0.001.

To further establish the association between TREM2 and renal fibrosis in obstructive nephropathy, UUO surgery was applied in WT mice (Figure 2A). At postoperative Day 14, obstructed kidneys exhibited marked hydronephrotic changes with cortical thinning (Figure 2B). Histological analysis revealed tubular epithelial necrosis with luminal cast formation and interstitial collagen deposition in obstructed kidneys after UUO (Figure 2C). Masson trichrome staining confirmed significantly exacerbated fibrosis in obstructed kidneys (Figure 2C). IHC staining demonstrated substantial CD68^+^ macrophage infiltration within the renal interstitium of UUO obstructed kidneys, indicating macrophage accumulation in fibrotic regions (Figure 2C). Concurrently, TREM2^+^ cells were significantly increased and similarly localised to the renal interstitium (Figure 2C).

IF staining of renal sections from UUO mice revealed a significant increase in the number of CD68^+^ cells and concomitant TREM2 upregulation in obstructed kidneys. Critical co‐localization of TREM2 with CD68^+^ macrophages was observed (Figure 2D). Spearman correlation confirmed a strong positive association between the number of TREM2^+^CD68^+^ cells and α‐SMA positive area (*r*_s_ = 0.9218, *p* < 0.0001) (Figure 2E).

**FIGURE 3:**
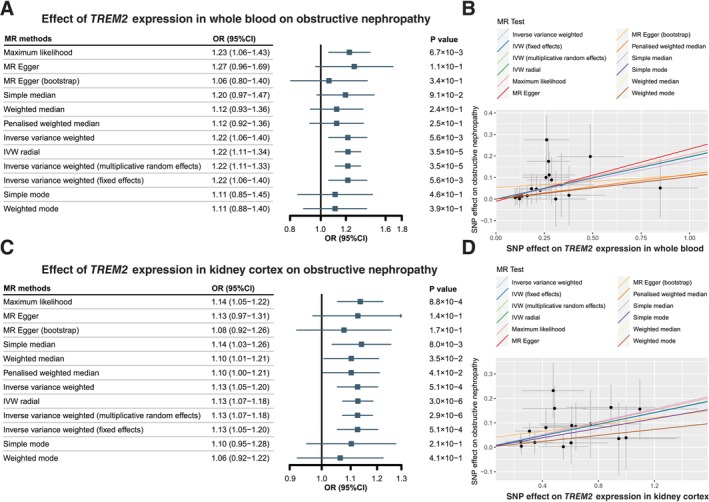
The relationship between TREM2 genetic variation and obstructive nephropathy. (A) The OR estimates and their 95% CI (confidence interval) for the effect of *TREM2* expression in whole blood on obstructive nephropathy, as assessed by various Mendelian Randomization (MR) methods. The boxes in the figure represent the HR corresponding to the use of different measurement methods. HR, hazard ratio; IVW, inverse variance weighted. (B) Scatterplots of the causal relationships between *TREM2* expression in whole blood and obstructive nephropathy. The *X*‐axis represents the genetic tool‐*TREM2* expression in whole blood association and the *Y*‐axis represents the genetic tool‐obstructive nephropathy association. The slope of each line corresponds to the estimated MR effect of each method. (C) The OR estimates and their 95% CI for the effect of *TREM2* expression in kidney cortex on obstructive nephropathy, as assessed by various MR methods. (D) Scatterplots of the causal relationships between *TREM2* expression in kidney cortex and obstructive nephropathy.

To assess the relationship between TREM2 genetic variation and obstructive nephropathy, we established a large population‐based cohort from the UKB and performed GWAS. The cohort construction strategy is detailed in the Methods section. The most strongly associated variants, with *p* values less than 8.6e‐05 and odds ratios up to 2.5, are listed in the Figure S1. Subsequent eQTL Mendelian randomization analyses demonstrated a significant positive association between TREM2 expression in blood and the risk of obstructive nephropathy. This finding was robust across multiple MR approaches, including Maximum likelihood, Simple median, Penalised weighted median and IVW models (Figure 3A,B; Table S4). In contrast, no significant effect was observed in kidney cortex tissue, where the association between TREM2 expression and obstructive nephropathy was negative but not statistically significant (Figure 3C,D; Table S5).

### 2.3 TREM2 Inhibitor Attenuated UUO Induced Renal Fibrosis in Mice

**FIGURE 4:**
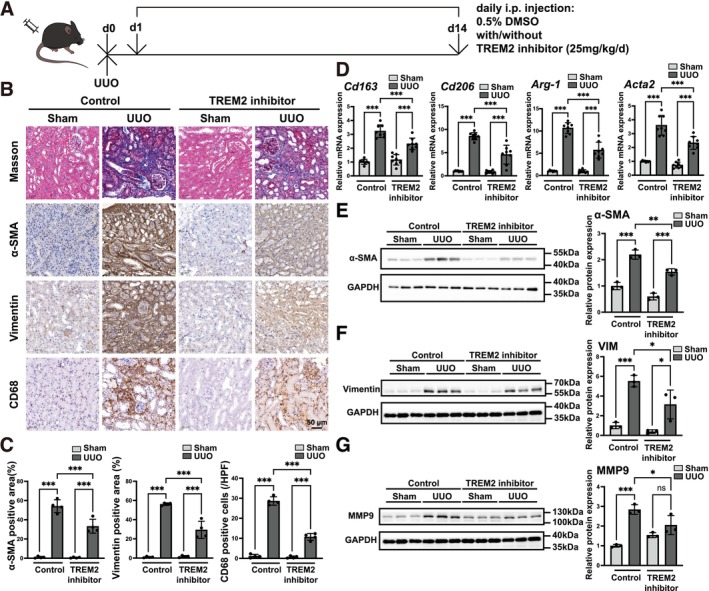
TREM2 inhibitor attenuated renal fibrosis in vivo. (A) Schematic graph to show the time point of TREM2 inhibitor or vehicle. The drug was daily intraperitoneal injected after UUO. (B) Representative Masson and IHC images of α‐SMA, Vimentin and CD68 of Control and TREM2 inhibitor treated‐group. Bars = 50 μm. (C) α‐SMA, Vimentin positive region and the number of CD68^+^ cells were significantly upregulated after UUO surgery, whereas inhibitor treatment attenuated this upregulation (*n* = 4). (D) Renal *Cd163*, *Cd206* and *Acta2* mRNA expression in the indicated mouse groups were measured by RT‐qPCR (*n* = 8). (E–G) Renal α‐SMA, Vimentin and MMP9 protein expression in the indicated mouse groups were measured by Western blot; the results were subjected to quantitative analysis (*n* = 3). Data are expressed as the mean ± SD. *p < 0.05; **p < 0.01; ***p < 0.001.

In order to assess the role of TREM2 in renal fibrosis, we established a UUO model in WT mice and treated them daily with the TREM2 inhibitory peptide sequence IA9 or vehicle (Figure 4A). The treatment group exhibited markedly less histologic damage (glomerular damage, collagen deposition) and reduced renal fibrosis compared to controls (Figure 4B,C). Furthermore, the TREM2 inhibitor substantially suppressed macrophage recruitment, with a decreased number of CD68^+^ cells in obstructed kidneys compared to controls (Figure 4B,C).

To investigate the effects of TREM2 inhibitor on fibrotic and macrophage polarisation, qPCR analysis revealed that TREM2 inhibition significantly downregulated mRNA levels of M2 macrophage markers (*Cd163*, *Cd206*, *Arg‐1*) and fibrosis‐related indicators *Acta2* (Figure 4D). Furthermore, the TREM2 inhibitor suppressed the protein expression of fibrosis markers α‐SMA, Vimentin and Matrix Metalloproteinase 9 (MMP9), as evidenced by Western blot (WB) and subsequent quantitative analysis (Figure 4E–G).

### 2.4 TREM2 Deficiency Prevented UUO‐Induced Renal Fibrosis In Vivo

**FIGURE 5:**
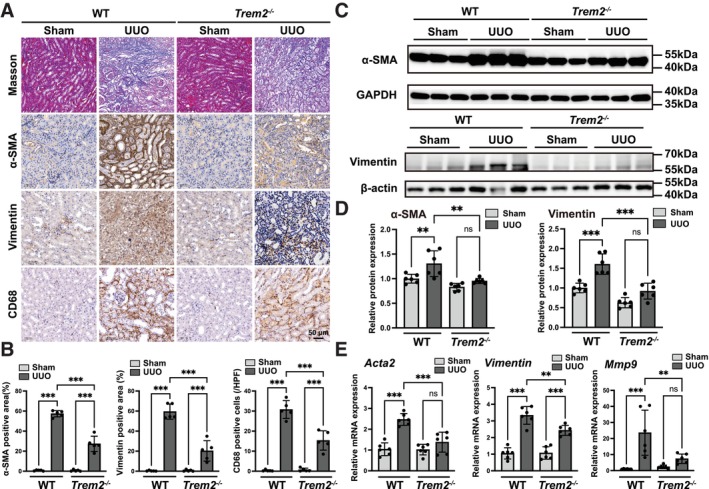
Global TREM2 deletion mitigated renal fibrosis in the 14 day‐UUO mouse model. (A, B) Representative images and quantifications of Masson and IHC staining of α‐SMA, Vimentin and CD68 in the kidney from different groups of mice (*n* = 5). Bars = 50 μm. (C, D) Representative Western blot and quantification of α‐SMA and Vimentin protein expression in the indicated mouse groups (*n* = 6). (E) Renal mRNA expression of *Acta2, Vimentin* and *Mmp9* in the indicated mouse groups was measured by RT‐qPCR (*n* = 6). Data are expressed as the mean ± SD. *p < 0.05; **p < 0.01; ***p < 0.001.

To further confirm the inhibitory effect of TREM2 deficiency on renal fibrosis, we established UUO models in *Trem2* ^*−/−*^ and WT mice. Mice were sacrificed at day 14 after the UUO procedure and the renal specimens were harvested for analysis. Masson trichrome staining revealed significantly reduced interstitial collagen deposition in obstructed kidneys of *Trem2* ^*−/−*^ compared to WT mice (Figure 5A,B). Despite UUO‐induced upregulation of α‐SMA and Vimentin in obstructed kidneys, *Trem2* ^*−/−*^ mice exhibited significantly suppressed fibrosis progression. IHC demonstrated substantial CD68^+^ cell infiltration in UUO kidneys, with significantly less pronounced CD68^+^ cell accumulation in *Trem2* ^*−/−*^ versus WT mice (Figure 5A,B). WB and qPCR analyses consistently demonstrated that global *Trem2* deletion suppressed renal fibrosis, as shown by significantly reduced protein (α‐SMA, Vimentin) and mRNA (*Acta2*, *Mmp9* and *Vimentin*) levels of key fibrotic markers in obstructed kidneys compared to WT mice (Figure 5C–E).

**FIGURE 6:**
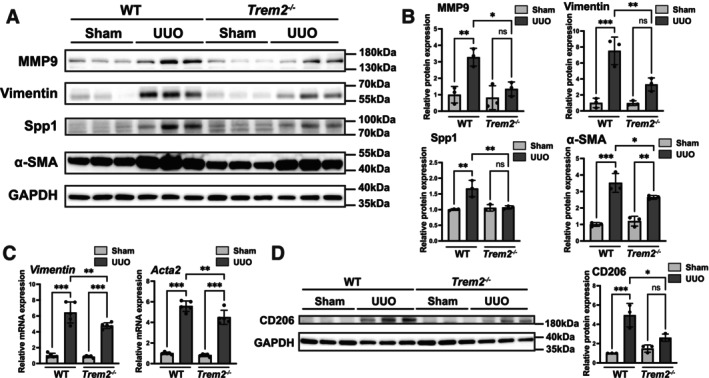
Extended 21‐day UUO experiments demonstrated that TREM2 deficiency suppressed renal fibrosis. (A, B) Representative Western blot and quantification of MMP9, Vimentin, Spp1, and α‐SMA protein expression in the indicated mouse groups (*n* = 3). (C) Renal mRNA expression of *Vimentin* and *Acta2* in the indicated mouse groups was measured by RT‐qPCR (*n* = 5). (D) Renal CD206 protein expression was measured by Western blot (*n* = 3). Data are expressed as the mean ± SD. *p < 0.05; **p < 0.01; ***p < 0.001.

Extended‐duration 21‐day UUO modelling was established in WT and *Trem2* ^*−/−*^ mice to further evaluate the temporal impact of TREM2 on renal fibrosis. WB and qPCR analyses demonstrated significantly attenuated renal fibrosis in *Trem2* ^*−/−*^ mice compared to WT mice (Figure 6), consistent with the observations from the 14‐day UUO model.

### 2.5 *Trem2* Deletion Attenuated GFR Decline in UUO Mice

**FIGURE 7:**
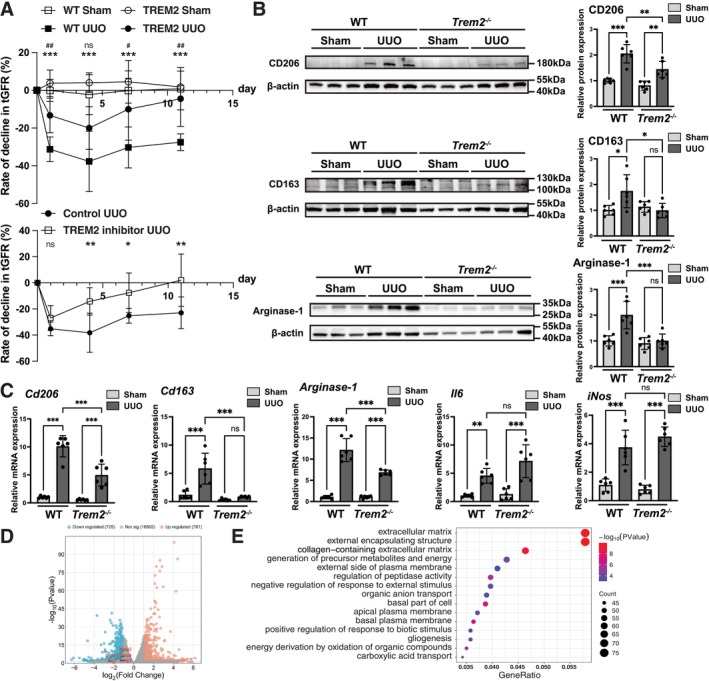
*Trem2* deletion attenuated GFR decline and inhibited macrophage M2 polarisation in UUO mice. (A) Dynamic changes in the decline rate of tGFR at different time points after UUO or sham surgery in WT and *Trem2* ^*−/−*^ mice. *Versus WT Sham with WT UUO. ^#^Versus WT UUO with *Trem2* ^*−/−*^ UUO. Dynamic changes in the decline rate of tGFR in TREM2 inhibitor and vehicle. (B) Representative Western blot and quantification of CD206, CD163 and Arginase‐1 protein expression in the indicated mouse groups (*n* = 6). (C) Renal mRNA expression of *Cd206*, *Cd163*, *Arginase‐1*, *Il6* and *iNos* in the indicated mouse groups (*n* = 6). (D) Gene expression difference of WT UUO and *Trem2* ^*−/−*^ UUO mice. (E) Gene ontology (GO) enrichment analysis of WT UUO and *Trem2* ^*−/−*^ UUO mice. The highly enriched categories include the extracellular matrix (ECM), external encapsulating structure and collagen‐containing extracellular matrix. Data are expressed as the mean ± SD. *p < 0.05; **p < 0.01; ***p < 0.001. ^*#*^ *p* < 0.05; ^*##*^ *p* < 0.01; ^*###*^ *p* < 0.001.

Glomerular filtration rate (GFR) was measured using the Transdermal Mini GFR Monitor system (MediBeacon GmbH, Mannheim, Germany) [22]. In WT UUO mice, GFR declined significantly at postoperative day 1 and subsequently stabilised at levels below sham‐operated controls (Figure 7A). *Trem2* ^−/−^ UUO mice similarly exhibited sustained GFR reduction from day 1 onward (Figure 7A). Critically, *Trem2* ^−/−^ mice demonstrated attenuated GFR decline relative to WT mice (Figure 7A), indicating that global *Trem2* knockout mitigated obstructive hydronephrosis‐induced renal functional impairment. Similarly, we found that the TREM2 inhibitor‐treated group exhibited a significantly lower degree of GFR decline compared to the control group, demonstrating that TREM2 inhibition also alleviates UUO‐induced renal function impairment (Figure 7A).

### 2.6 *Trem2* Deficiency Inhibited Macrophage M2 Polarisation in Kidneys of UUO Mice

To delineate the mechanistic role of TREM2 in renal fibrosis via macrophage immunomodulation, macrophage polarisation markers were assessed at mRNA and protein levels in kidneys from *Trem2* ^−/−^ and WT UUO mice. WB and qPCR analysis revealed that the UUO‐induced upregulation of M2 markers was significantly attenuated in *Trem2* ^−/−^ mice compared to WT mice at both protein and mRNA levels (Figure 7B,C). Notably, no statistically significant differences emerged in mRNA expression of M1 markers (*iNos*, *Il6*) between *Trem2* ^*−/−*^ and WT obstructed kidneys (Figure 7C).

RNA‐seq was conducted on kidneys from WT UUO and *Trem2* ^*−/−*^ UUO mice. Gene ontology (GO) enrichment analysis indicated that a series of fibrosis‐related genes, including those associated with the ECM, external encapsulating structure and collagen‐containing extracellular matrix, were significantly downregulated in *Trem2* ^*−/−*^ mice (Figure 7D,E).

### 2.7 *Trem2* Deletion Suppressed Profibrotic Activation via Impaired M2 Polarisation in IL‐4 and IL‐13‐Stimulated BMDMs

**FIGURE 8:**
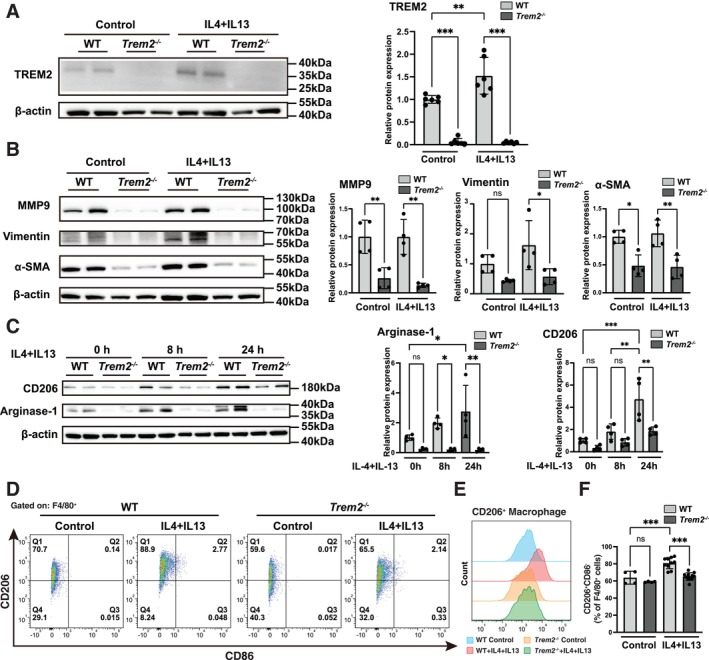
*Trem2* deletion suppressed fibrogenesis by impairing IL‐4/IL‐13 induced M2 polarisation in BMDMs. (A) TREM2 protein expression in WT and *Trem2* ^*−/−*^ BMDMs with or without IL‐4/IL‐13 stimulation was measured by Western blot (*n* = 6). (B) Representative Western blot and quantification of MMP9, Vimentin, and α‐SMA protein expression in the indicated groups (*n* = 4). (C) CD206 and Arginase‐1 protein expression in WT and *Trem2* ^*−/−*^ BMDMs after 0 h, 8 h, 24 h IL‐4/IL‐13 stimulation were measured by Western blot (*n* = 4). (D) The proportions of M2 phenotype macrophages (CD86^−^CD206^+^) with or without IL‐4/IL‐13 stimulation (gated on F4/80^+^ cells) were detected by flow cytometry assay in WT and *Trem2* ^*−/−*^ BMDMs. (E, F) Representative histograms of CD206^+^ macrophages across experimental groups and statistical analysis of M2 polarisation percentage in BMDMs (*n* = 4–11). Data are expressed as the mean ± SD. *p < 0.05; **p < 0.01; ***p < 0.001.

To elucidate how TREM2 regulates macrophage pro‐fibrotic mechanisms, we isolated BMDMs from femoral and tibiae of WT and *Trem2* ^−/−^ mice. Differentiated BMDMs were stimulated with IL‐4 and IL‐13 for analysis. WB confirmed IL‐4 and IL‐13 induced TREM2 upregulation in WT BMDMs, whereas TREM2 protein was absent in *Trem2* ^−/−^ BMDMs (Figure 8A). Subsequent analysis of α‐SMA, MMP9 and Vimentin protein levels post IL‐4 and IL‐13 stimulation demonstrated significantly higher expression of these fibrotic markers in WT BMDMs versus *Trem2* ^*−/−*^ BMDMs (Figure 8B).

IL‐4 and IL‐13 stimulation significantly increased the protein expression of CD206 and Arginase 1 (ARG‐1) in WT BMDMs in a time‐dependent manner, while this response was markedly attenuated in *Trem2* ^−/−^ BMDMs (Figure 8C). These data indicated that *Trem2* deletion inhibited IL‐4 and IL‐13‐induced M2 polarization of BMDMs in vitro.

Moreover, this hypothesis was verified by flow cytometry. Following IL‐4 and IL‐13 stimulation, the M2 macrophage (CD206^+^CD86^−^) ratio increased in both WT and *Trem2* ^*−/−*^ BMDMs, though the increase was significantly lower in *Trem2* ^*−/−*^ BMDMs, confirming that *Trem2* deficiency suppressed M2 polarization in BMDMs (Figure 8D–F).

### 2.8 TREM2 Deficiency Regulated β‐Catenin Signalling Pathway

**FIGURE 9:**
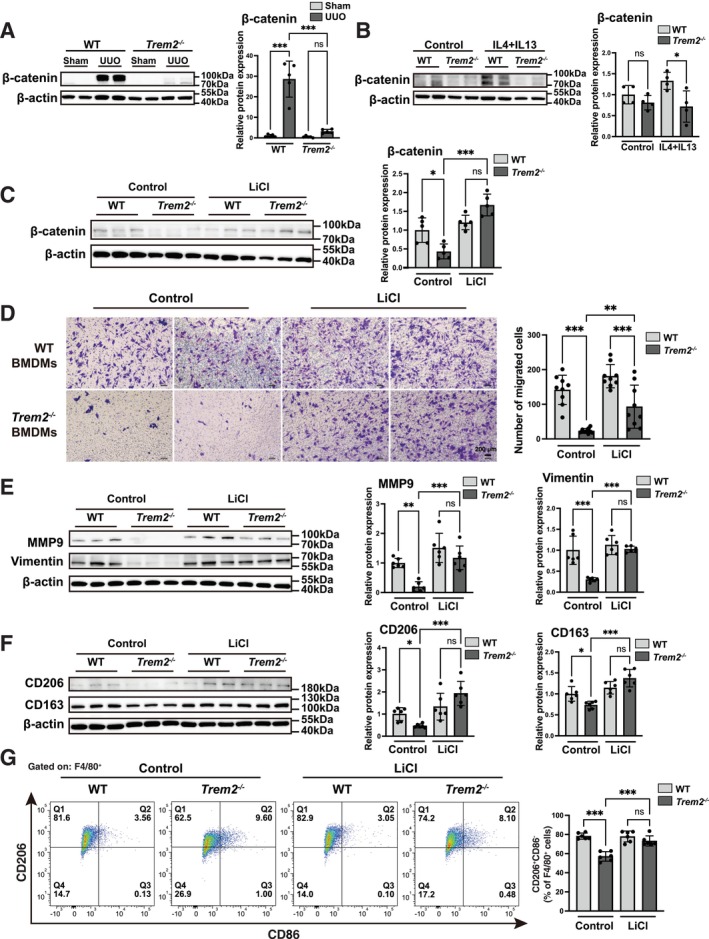
TREM2 deficiency regulated β‐catenin signalling pathway. (A) β‐catenin protein expression in WT and *Trem2* ^*−/−*^ UUO mice (*n* = 5). (B) β‐catenin protein expression in WT and *Trem2* ^*−/−*^ BMDMs with or without IL‐4/IL‐13 stimulation (*n* = 4). (C) β‐catenin protein expression in WT and *Trem2* ^*−/−*^ BMDMs with or without LiCl treatment (*n* = 5). (D) Transwell assay assessing migration of BMDMs from WT and *Trem2* ^*−/−*^ mice with or without LiCl treatment (*n* = 9). Bars = 200 μm. (E) Representative Western blot and quantification of MMP9 and Vimentin protein expression (*n* = 6). (F) Representative Western blot and quantification of CD206 and CD163 protein expression (*n* = 6). (G) The proportions of M2 phenotype macrophages (CD86^−^CD206^+^) were detected by flow cytometry assay in WT and *Trem2* ^*−/−*^ mice with or without LiCl treatment (*n* = 6). Data are expressed as the mean ± SD. *p < 0.05; **p < 0.01; ***p < 0.001.

To determine whether TREM2 exerted a regulatory effect on macrophage polarisation through the β‐catenin signalling pathway, we assessed the β‐catenin protein expression in WT and *Trem2* ^*−/−*^ UUO mice. The results showed that β‐catenin was upregulated in obstructed kidneys of UUO mice, whereas *Trem2* ^*−/−*^ suppressed this upregulation (Figure 9A). In vitro, β‐catenin protein levels were substantially reduced in *Trem2* ^*−/−*^ BMDMs (Figure 9B). We next evaluated the effects of LiCl on *Trem2* ^*−/−*^ BMDMs. Treatment with LiCl successfully increased β‐catenin protein in *Trem2* ^*−/−*^ BMDMs (Figure 9C). Furthermore, the LiCl‐mediated β‐catenin stabilisation rescued the impaired macrophage migration (Figure 9D) and elevated the expression of M2 markers (CD206, CD163) and fibrotic proteins (Vimentin, MMP9) in *Trem2* ^*−/−*^ BMDMs (Figure 9E,F). Consistent with these findings, flow cytometry results further confirmed that LiCl stimulation significantly increased the ratio of M2 macrophages (CD206^+^CD86^−^) in *Trem2* ^*−/−*^ BMDMs (Figure 9G).

## 3 Discussion

TREM2 is an immune receptor expressed on myeloid cells that regulates inflammatory responses. Importantly, the study demonstrated that TREM2 promoted renal fibrosis in obstructive nephropathy. Accordingly, we observed elevated TREM2 expression in human obstructed kidneys, where the number of TREM2^+^CD68^+^ cells showed a positive correlation with fibrosis severity. *Trem2* ^*−/−*^ mice exhibited reduced M2 macrophage infiltration, attenuated renal fibrosis and better‐preserved renal function post‐UUO, indicating a renoprotective potential of TREM2 deficiency. Mechanistically, *Trem2* ^*−/−*^ BMDMs displayed suppressed β‐catenin signalling, impaired M2 polarisation and diminished production of fibrotic proteins. Critically, TREM2 inhibitor administration in WT UUO mice significantly attenuated renal fibrosis and GFR decline. Collectively, the study established that TREM2 promotes renal fibrosis by activating the β‐catenin signalling pathway, driving M2 macrophage polarisation, positioning it as a promising therapeutic target for antifibrotic strategies.

The role of TREM2 in macrophages during inflammatory and fibrotic processes has garnered significant interest in recent years. Substantial evidence indicates that TREM2 plays important roles in non‐alcoholic liver fibrosis, pulmonary fibrosis and hepatocellular carcinoma, yet its functions exhibit marked context‐dependence across different cell types and organs [9, 10, 23]. Our findings supported a profibrotic function for TREM2 in obstructive nephropathy. Specifically, we have discovered that TREM2 plays a pivotal role in driving macrophage polarisation toward the M2 phenotype. Contrastingly, a study demonstrated that TREM2 deficiency exacerbated inflammation and fibrosis on postoperative day 3 and 7 in the UUO model [14]. This discrepancy may be attributed to temporal heterogeneity, while renal fibrosis typically becomes more pronounced 2–3 weeks after surgery, aligning with our observations and previous findings [13]. Notably, the 3‐ and 7‐day UUO models likely emphasise the acute inflammatory and initial fibrotic responses during early injury. At this stage, TREM2 deficiency may more pronouncedly suppress macrophage anti‐inflammatory functions, thereby exacerbating tissue damage. Unlike their discovery that TREM2 deficiency increases M1/M2 macrophage infiltration and exacerbates renal injury, our work demonstrated suppressed M2 macrophage infiltration following *Trem2* deletion. The discrepancy underscores that the potential time‐dependent nature of TREM2 functions, procedural variations in surgical modelling and differences in husbandry conditions may significantly influence ultimate inflammatory and fibrotic phenotypes. Moreover, this study dynamically monitored post‐UUO renal function changes in mice and conducted TREM2 inhibitor administration experiments to assess its therapeutic potential, thereby yielding more reliable results.

Mechanistically, TREM2 may modulate macrophage phenotypic switching, proliferation and migration through β‐catenin signalling pathway. β‐catenin signalling is known to drive renal interstitial fibrosis by promoting fibroblast‐to‐myofibroblast transition [24]. Established studies have indicated that TREM2 or its adaptor molecule DAP12 activates β‐catenin signalling in M‐CSF‐stimulated myeloid cells [25]. Furthermore, TREM2 enhances macrophage M2 proliferation through β‐catenin upregulation [26]. In obstructive nephropathy, we demonstrated that *Trem2* ^−/−^ significantly downregulated β‐catenin pathway‐related genes and suppressed pathological upregulation of β‐catenin protein levels, indicating TREM2 promotes macrophage M2 polarisation and migration through β‐catenin pathway activation. In vitro experiments further validated this mechanism, demonstrating that *Trem2* ^−/−^ inhibited IL‐4 and IL‐13‐induced β‐catenin upregulation in BMDMs. Critically, LiCl effectively reversed this deficit, restoring β‐catenin protein expression in *Trem2* ^*−/−*^ BMDMs, concomitant with enhanced M2 polarisation and upregulated profibrotic proteins production. Collectively, our study demonstrated that TREM2 activates the β‐catenin signalling pathway to stabilise macrophage state, enhance migration, drive M2 polarisation and ultimately accelerates renal fibrosis in obstructive nephropathy.

Classical methods to measure renal function are mostly based on elevated Scr and/or reduced urine output. However, these parameters are susceptible to extra‐renal confounding factors and typically require anaesthesia, which may alter renal hemodynamics and compromise accuracy [27]. To address these limitations, the study employed tGFR monitoring for dynamic renal function evaluation. Notably, studies have reported that UUO mice exhibited markedly decreased tGFR without significant changes in plasma creatinine [28] and that all renal injury biomarkers lagged behind real‐time GFR changes during AKI progression or recovery [27], highlighting the early‐detection value of tGFR. Moreover, the non‐invasive technique enables serial measurements in individual mice across multiple timepoints, eliminating the need for repeated blood sampling and anaesthesia, thereby minimising procedural confounders, enhancing data reliability, improving animal welfare and reducing overall animal usage.

Based on the finding from *Trem2* ^*−/−*^ mice that TREM2 plays a profibrotic role, we directly assessed its therapeutic potential by TREM2 inhibitor administration in vivo. Previous studies have demonstrated that TREM2 inhibitors reduce pro‐inflammatory cytokine release, alleviating joint inflammation and damage [29]. Additionally, TREM2 inhibitors reprogram tumour‐associated macrophages composition in the tumour microenvironment [30]. Collectively, TREM2 inhibitors play a critical role in modulating inflammatory responses. Crucially, our work revealed that TREM2 protein inhibitors effectively suppress macrophage M2 polarisation and profibrotic protein production, significantly attenuating renal interstitial fibrosis in UUO models. These findings indicate that targeting macrophage TREM2 represents a highly promising novel strategy for treating renal fibrosis in obstructive nephropathy. Given that renal fibrosis is a core pathological change in progression of chronic kidney diseases and considering the current absence of effective and specific clinical antifibrotics, our study provides experimental evidence supporting the development of TREM2‐targeted therapies with potential for clinical translation.

Although our study demonstrates a clear role for TREM2 in renal fibrosis using global *Trem2*‐knockout mice, this approach has inherent limitations. The global knockout model cannot distinguish the specific contribution of macrophage‐expressed TREM2 from its functions in other myeloid cell populations (e.g., dendritic cells). Furthermore, potential developmental compensatory mechanisms cannot be ruled out. To unequivocally establish the cell‐specific mechanisms of TREM2 in renal fibrosis, future work utilising conditional genetic models will be required.

## 4 Conclusion

In summary, this study demonstrated that TREM2 promotes obstructive nephropathy progression by activating the β‐catenin signalling pathway, driving M2 macrophage polarisation, migration and fibrogenic gene expression. TREM2 deficiency reduces macrophage recruitment and infiltration in fibrotic tissues, inhibits M2 polarisation, significantly attenuates renal fibrosis and may confer renoprotective effects. Targeted inhibition of TREM2 thus represents a promising novel strategy against renal fibrosis.

## 5 Methods

### 5.1 Human Kidney Samples

Written informed consent was acquired from adult participants (≥ 18 years) and legal guardians of minors. Patient privacy was safeguarded through full anonymization of all data. Kidney tissue specimens were stratified according to predefined criteria: (1) Renal Fibrosis Cohort: comprising surgically resected renal parenchyma from urethral obstruction patients; (2) Control Cohort: consisting of histologically normal parenchymal tissue obtained from tumour‐adjacent regions without prior chemotherapy or trauma resection sites. Exclusionary parameters eliminated specimens from individuals with polycystic or dysplastic kidney disorders, secondary fibrosis, immunodeficiency states, viral hepatitis, or associated comorbidities, as well as cases lacking complete clinical documentation. Comprehensive subject characteristics are catalogued in Table S1.

### 5.2 Animals

All eight‐week‐old C57BL/6J (WT) male mice were purchased from Hangzhou Qizhen Laboratory Animal Technology Co. Ltd. Male *Trem2* ^*−/−*^ (C57BL/6J) mice were kindly gifted by Dr. Xiangming Fang (Department of Anesthesiology, The First Affiliated Hospital, Zhejiang University School of Medicine, Hangzhou, China). All animals were housed under specific pathogen‐free (SPF) conditions at the Zhejiang Chinese Medical University Laboratory Animal Research Center. The facility maintained constant temperature and humidity with ad libitum access to food and water under a 12/12‐h dark–light cycle. All experimental procedures involving animal husbandry, modelling and handling strictly complied with institutional guidelines for laboratory animal management and ethical requirements.

### 5.3 UUO Model

The UUO model was established as previously described [31]. Briefly, mice were anaesthetised via inhalation of isoflurane followed by intraperitoneal injection of pentobarbital sodium (40 mg/kg). The left ureter was exposed and ligated using 3–0 silk sutures at two distinct points with a 1 mm interval between the ligatures. Sham‐operated controls underwent identical procedures without ureteral ligation. At postoperative day 14 or 21, mice were euthanized and the kidneys were harvested. All surgical procedures were conducted independently by a single experienced operator blinded to genotype and experimental grouping, with awareness limited to animal identification codes.

### 5.4 IA9 Treatment

Eight‐week‐old male WT mice underwent UUO modelling or sham surgery and randomization into control and treatment groups. For 14 consecutive days post‐surgery, the treatment group received daily intraperitoneal injections of the TREM2 inhibitor IA9 (Weichen Biotechnology, China) at 25 mg/kg/day [29], while the control group received equivalent volumes of 0.5% DMSO‐saline solution. The animals were euthanized 14 days after surgery and the kidney tissues were collected for further analysis.

### 5.5 Transdermal Glomerular Filtration Rate Measurement

Transdermal GFR measurement was done as described previously [19, 32]. In short, dorsolateral skin (paraspinal region avoided) was clipped and depilated to expose bare skin and a miniaturised fluorescence detector (MediBeacon GmbH, Mannheim, Germany) was mounted onto the depilated animals' back. After securing the assembled device to the skin, mice were individually housed in quiet cages for 5–10 min. Subsequently, 70 mg/kg of FITC‐sinistrin (MediBeacon GmbH, Mannheim, Germany) was injected via the orbital plexus. The device was removed 1–2 h post‐injection and the data were analysed using MB Lab/MB Studio software (MediBeacon GmbH, Mannheim, Germany).

### 5.6 Cell Culture and Treatment

Mouse bone marrow‐derived macrophages (BMDMs) were isolated from tibiae and femora of WT and *Trem2* ^*−/−*^ mice by flushing with DMEM. Cells were cultured for 7–8 days in the complete medium High‐glucose Dulbecco's modified Eagle's medium (DMEM; Gibco, China) containing 10% FBS (Procell, China), 1% penicillin/streptomycin solution (Biosharp, China) and 20 ng/mL macrophage colony‐stimulating factor (M‐CSF; Pricella, China). At experimental endpoints, cells were harvested with or without 20 ng/mL interleukin‐4 (IL‐4) and 20 ng/mL interleukin‐13 (IL‐13) stimulation. For rescue experiments, WT or *Trem2* ^*−/−*^ BMDMs were treated with 5 mM LiCl (a GSK‐3 inhibitor that can stabilise β‐catenin) for 24 h [33].

### 5.7 Histology

Human and murine kidney sections (4 μm) were prepared from paraffin‐embedded tissues. After deparaffinisation, sections underwent routine Haematoxylin and eosin (H&E) staining and Masson's trichrome staining (MTS). For IHC, deparaffinised sections were rehydrated, followed by sequential blocking of endogenous peroxidase with 3% hydrogen dioxide (30 min, RT) and non‐specific binding with 1% BSA (30 min, RT). Primary antibodies were applied overnight at 4°C, followed by 40 min RT incubation with secondary antibodies. Diaminobenzidine (DAB) was used for visualisation. For IF, sections were incubated with primary antibodies overnight at 4°C and fluorophore‐conjugated secondary antibodies for 1 h at RT, followed by DAPI counterstaining. Slides were imaged using a fluorescence microscope (Leica company, Germany). For the quantitative analysis, 10 × 400 fields were randomly selected in the cortical area from each kidney section. Antibody details are listed in Table S2.

### 5.8 Western Blotting Assay

Kidney tissues and cells were lysed in RIPA buffer (Meilunbio, China) supplemented with phosphatase inhibitors, protease inhibitors and PMSF (Meilunbio, China). Equal protein quantities from all samples were separated by SDS‐PAGE (Servicebio, China) and transferred to methanol‐activated PVDF membranes (Millipore, USA). After blocking with 5% skim milk for 2 h at RT, membranes were incubated overnight at 4°C with primary antibodies followed by 1 h incubation with HRP‐conjugated secondary antibodies at room temperature. Protein bands were visualised by chemiluminescent detection. All antibodies are catalogued in Table S2.

### 5.9 Quantitative Real‐Time PCR

Total RNA was extracted from kidney tissues and BMDMs using the Easy RNA Extraction Kit (Easydo Biotech, China). cDNA synthesis was performed with the PrimeScript RT reagent Kit (Cat. #RR036A, Takara, Japan). β‐actin served as the endogenous control and relative gene expression was calculated using the 2^−ΔΔCt^ method. All primer sequences are provided in Table S3.

### 5.10 Transwell Migration Assay

Transwell migration assays were performed in 24‐transwell chambers (5 um pores, Corning, Cat #: 3421) according to the manufacturer's instructions. Briefly, BMDMs were serum‐starved for 3 h before seeding 100,000 cells in the upper chamber with serum‐free DMEM. The lower chamber was filled with 750 μL complete DMEM supplemented with 10% FBS and 20 ng/mL M‐CSF, and the transwell plates were incubated in 5% CO_2_ for 24 h. To stabilise β‐catenin, cells were treated with 5 mM LiCl or vehicle in the upper and lower chambers. Following migration, transwell inserts were fixed with 4% glutaraldehyde for 10 min at RT and stained with 0.1% crystal violet for 20 min at RT after PBS washes. Images were taken after the insert completely dried.

### 5.11 Flow Cytometry

Flow cytometric analysis was carried out according to standard procedures [34]. Briefly, BMDMs were suspended in PBS and blocked for unspecific binding with rat anti‐mouse CD16/CD32 antibody (Cat# 553141; BD Biosciences) and incubated with BV421 rat anti‐mouse F4/80 (Cat# 565411; BD Biosciences), PE anti‐mouse CD86 (Cat# 159204; BioLegend), and APC anti‐mouse CD206 (Cat# 141708; BioLegend) antibodies or isotype controls at 4°C for 40 min in a dark place. The cells were then washed in PBS and detected by a flow cytometry (BD FACSCelesta, USA). The results were analysed using FlowJo software version 10.9.0 (FlowJO LLC, Ashland, OR, USA).

### 5.12 Statistics

All statistical analyses were performed using GraphPad Prism Version 10.1.2 software. Data were represented as mean ± standard deviation (SD) from at least 3 independent experiments. Differences between two unpaired groups were assessed by Student's *t*‐test. Comparisons across more than two unpaired groups used one‐way ANOVA with Tukey's post hoc test. Correlation analyses employed Spearman's rank correlation. Statistical significance was defined as *p* < 0.05.

### 5.13 Study Approval

All research procedures rigorously adhered to the Declaration of Helsinki and received formal approval from the Clinical Ethics Committee of the Children's Hospital, Zhejiang University School of Medicine (Ethics No. 2024‐IRBAL‐0208). Written informed consent was obtained from all the patients and their guardian. The genetic association exploration was approved by the North West Multicenter Research Ethics Committee (approval number: 11/NW/0382).

## Author Contributions

J.W. designed the research. J.W. and X.Y. supervised all investigations. J.W., Z.L., G.D., M.Y. collected and analysed the clinical data. Z.L., G.D., M.Y., Z.Y. and Y.Z. performed experiments and analysed data. J.W., Z.L., G.D. and T.C. wrote the manuscript. Z.L. and G.D. prepared figures and tables. All authors provided critical reviews of the manuscript.

## Funding

This work was supported by the National Natural Science Foundation of China (823717090), the Huadong Medicine Joint Fund of Zhejiang Provincial Natural Science Foundation of China (LHDMZ25H050002).

## Conflicts of Interest

The authors declare no conflicts of interest.

## Supporting information


**Data S1:** cpr70192‐sup‐0001‐supinfo.pdf.
**Table S1:** Background information on the obstructive nephropathy patients and normal controls.
**Table S2:** The list of antibodies used in this study.
**Table S3:** The list of primers for RT‐qPCR.
**Table S4:** Mendelian Randomization analyses estimating the TREM2 expression in whole blood.
**Table S5:** Mendelian Randomization analyses estimating the TREM2 expression in kidney cortex.
**Figure S1:** Results of leave‐one‐out method sensitivity analysis and funnel plots. (A) Leave‐one‐out analysis of single nucleotide polymorphisms (SNPs) associated with TREM2 expression in kidney cortex of obstructive nephropathy patients. (B) Funnel plots for IVW and MR‐Egger methods assessing the causal effect of TREM2 expression in kidney cortex on obstructive nephropathy. (C) Leave‐one‐out analysis of SNPs associated with TREM2 expression in whole blood of obstructive nephropathy patients. (D) Funnel plots for IVW and MR‐Egger methods assessing the causal effect of TREM2 expression in whole blood on obstructive nephropathy.

## Data Availability

The data that support the findings of this study are available from the corresponding author upon reasonable request.
