# Supplementary material for: TREM2 in Macrophages Promotes Renal Fibrosis via Activation of β‐Catenin Signalling Pathway in Obstructive Nephropathy
Source: Cell Prolif. 2026 Mar 7;59(8):e70192. doi: 10.1111/cpr.70192 (PMC13325555; doi:10.1111/cpr.70192)
Supplement: Supplementary file 1 [file CPR-59-e70192-s001.pdf]

Supplementary Table 1. Background information on the obstructive nephropathy patients and normal controls

Obstructive nephropathy patients (n=24)

		**Number (n)**	**Percentage (%)**
Age(years)	<18	19	79.2%
	≥18	5	20.8%
Gender	Male	15	62.5%
	Female	9	37.5%
Reason of obstruction	UPJO*	13	54.2%
	Urinary tract stone	8	33.3%
	Tumor	3	12.5%
Degree of kidney fibrosis	Mild	6	25%
	Moderate	7	29.2%
	Severe	11	45.8%

*UPJO: Ureteropelvic Junction Obstruction

Normal controls (n=12)

		**Number (n)**	**Percentage (%)**
Age(years)	<18	4	33.3%
	≥18	8	66.7%
Gender	Male	5	41.7%
	Female	7	58.3%

Supplementary Table 2. The list of antibodies used in this study

Antibody	Provider	Fluorochrome	Source	Catalog
α-SMA	eBioscience	--	Mouse	ab2572996
Vimentin	Abcam	--	Rabbit	ab92547
MMP9	Proteintech	--	Rabbit	10375-2-AP
CD206	Proteintech	--	Rabbit	18704-1-AP
CD206	Genetex	--	Rat	GTX42263
CD163	Proteintech	--	Rabbit	16646-1-AP
CD163	ProMab	--	Mouse	31803
Arginase-1	Proteintech	--	Rabbit	16001-1-AP
GAPDH	Invitrogen	--	Mouse	ab10977387
β-actin	Invitrogen	--	Rabbit	ab2745831
TREM2	Abcam	--	Rabbit	ab318262
TREM2	Abcam	--	Rabbit	ab305103
CD68	Abcam	--	Mouse	ab955
CD68	Abcam	--	Rat	ab53444
β-catenin	Proteintech	--	Mouse	66379-1-lg
β-catenin	Proteintech	--	Rabbit	51067-2-AP
Spp1	Proteintech	--	Rabbit	22952-1-AP
CD16/CD32	BD Biosciences	--	Rat	553141
F4/80	BD Biosciences	BV421	Rat	565411
CD86	BioLegend	PE	Rat	159204
CD206	BioLegend	APC	Rat	141708

Supplementary Table 3. The list of primers for RT-qPCR

Gene	Direction	Sequence
Actin	Forward	5'-CAG CCT TCC TTC TTG GGT ATG-3'
	Reverse	5'-GGC ATA GAG GTC TTT ACG GAT G-3'
Acta2	Forward	5'-GTC CCA GAC ATC AGG GAG TAA-3'
	Reverse	5'-TCG GAT ACT TCA GCG TCA GGA-3'
Mmp9	Forward	5'-GCT GAC TAC GAT AAG GAC GGC A-3'
	Reverse	5'-TAG TGG TGC AGG CAG AGT AGG A-3'
Il-6	Forward	5'-GCC TTC TTG GGA CTG ATG CT-3'
	Reverse	5'-GCC ATT GCA CAA CTC TTT TCT CA-3'
Cd163	Forward	5'-AAT CAC ATC ATG GCA CAG GTC ACC-3'
	Reverse	5'-TCG TCG CTT CAG AGT CCA CAG G-3'
Cd206	Forward	5'-TGA TTG GTG GCA ATT CAC GAG AGG -3'
	Reverse	5'-AAC AGG CAG GGA AGG GTC AGT C-3'
iNos	Forward	5'-CAG CTG GGC TGT ACA AAC CTT-3'
	Reverse	5'-TGA AGC CAT TTT GGT GTT CTT-3'
Vimentin	Forward	5'-CAC TAG CCG CAG CCT CTA TTC-3'
	Reverse	5'-GTC CAC CGA GTC TTG AAG CA-3'
Arginase-1	Forward	5'-CTC CAA GCC AAA GTC CTT AGA G-3'
	Reverse	5'-AGG AGC TGT CAT TAG GGA CAT C-3'

Supplementary Table 4. Mendelian Randomization analyses estimating the TREM2 expression in whole blood

Outcome	Exposure	Method	Number of SNPs	Beta	SE	*P* value
Obstructive nephropathy	TREM2 expression in whole blood	Maximum likelihood	19	0.207363027	0.076419127	0.006657729
Obstructive nephropathy	TREM2 expression in whole blood	MR Egger	19	0.242842295	0.143157131	0.108055782
Obstructive nephropathy	TREM2 expression in whole blood	MR Egger (bootstrap)	19	0.059334591	0.141983305	0.337
Obstructive nephropathy	TREM2 expression in whole blood	Simple median	19	0.17903946	0.106081373	0.091458102
Obstructive nephropathy	TREM2 expression in whole blood	Weighted median	19	0.115103302	0.097149453	0.236093967
Obstructive nephropathy	TREM2 expression in whole blood	Penalised weighted median	19	0.115103302	0.099212862	0.245981586
Obstructive nephropathy	TREM2 expression in whole blood	Inverse variance weighted	19	0.195874057	0.070743078	0.005626165
Obstructive nephropathy	TREM2 expression in whole blood	IVW radial	19	0.197300619	0.047710503	3.54E-05
Obstructive nephropathy	TREM2 expression in whole blood	Inverse variance weighted (multiplicative random effects)	19	0.195874057	0.047366032	3.54E-05
Obstructive nephropathy	TREM2 expression in whole blood	Inverse variance weighted (fixed effects)	19	0.195874057	0.070743078	0.005626165
Obstructive nephropathy	TREM2 expression in whole blood	Simple mode	19	0.103893387	0.138179734	0.461847399
Obstructive nephropathy	TREM2 expression in whole blood	Weighted mode	19	0.103893387	0.117070584	0.386545357

Supplementary Table 5 Mendelian Randomization analyses estimating the TREM2 expression in kidney cortex.

Outcome	Exposure	Method	Number of SNPs	Beta	SE	*P* value
Obstructive nephropathy	TREM2 expression in kidney cortex	Maximum likelihood	16	0.127077857	0.038188852	0.000875923
Obstructive nephropathy	TREM2 expression in kidney cortex	MR Egger	16	0.118893863	0.075578236	0.138011518
Obstructive nephropathy	TREM2 expression in kidney cortex	MR Egger (bootstrap)	16	0.073241016	0.079428187	0.17
Obstructive nephropathy	TREM2 expression in kidney cortex	Simple median	16	0.130936831	0.049334025	0.007952315
Obstructive nephropathy	TREM2 expression in kidney cortex	Weighted median	16	0.097161645	0.046060707	0.034907841
Obstructive nephropathy	TREM2 expression in kidney cortex	Penalised weighted median	16	0.097161645	0.047496529	0.040790052
Obstructive nephropathy	TREM2 expression in kidney cortex	Inverse variance weighted	16	0.118105553	0.033989914	0.00051138
Obstructive nephropathy	TREM2 expression in kidney cortex	IVW radial	16	0.119053065	0.02550224	0.00000304
Obstructive nephropathy	TREM2 expression in kidney cortex	Inverse variance weighted (multiplicative random effects)	16	0.118105553	0.025255716	0.00000292
Obstructive nephropathy	TREM2 expression in kidney cortex	Inverse variance weighted (fixed effects)	16	0.118105553	0.033989914	0.00051138
Obstructive nephropathy	TREM2 expression in kidney cortex	Simple mode	16	0.097343528	0.074926977	0.213497824
Obstructive nephropathy	TREM2 expression in kidney cortex	Weighted mode	16	0.060908861	0.0720766	0.411355748

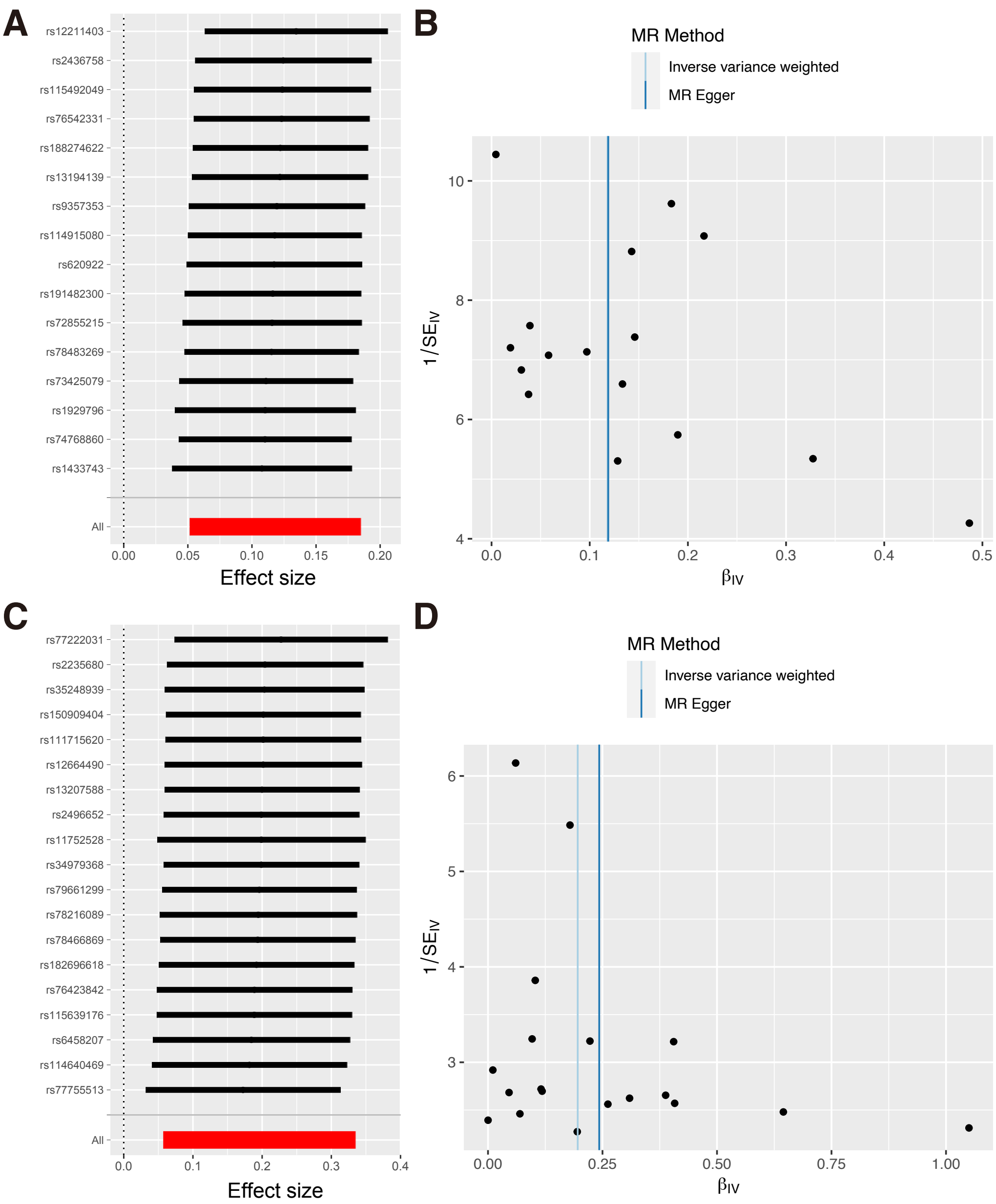

Figure S1. Results of leave-one-out method sensitivity analysis and funnel plots. (A) Leave-one-out analysis of single nucleotide polymorphisms (SNPs) associated with *TREM2* expression in kidney cortex of obstructive nephropathy patients. (B) Funnel plots for IVW and MR-Egger methods assessing the causal effect of *TREM2* expression in kidney cortex on obstructive nephropathy. (C) Leave-one-out analysis of SNPs associated with *TREM2* expression in whole blood of obstructive nephropathy patients. (D) Funnel plots for IVW and MR-Egger methods assessing the causal effect of *TREM2* expression in whole blood on obstructive nephropathy.

Supplementary Materials and Methods

Genetic Association Exploration

All participants in the UK Biobank (UKB) provided written informed consent prior to enrollment. Genotypic and clinical data were obtained from the UK Biobank under application number 105945. The analysis included unrelated individuals of genetically confirmed White British ancestry, selected from the total 503,317 UKB participants. Disease status was defined based on primary inpatient diagnoses, main death records, and principal operation codes, according to the International Classification of Diseases, Tenth Revision (ICD-10; field IDs: 41202, 40001). Participants diagnosed with codes N13, N20, N21, or N32.0 were classified as cases of obstructive nephropathy, while all remaining UKB participants served as controls. Genome-wide association studies (GWAS) were conducted on the UKB cohort using the UK BiLEVE Axiom and UK Biobank Axiom genotyping arrays. Mendelian randomization (MR) analyses were performed using the TwoSampleMR R package (version 0.6.4) and data from the Genotype-Tissue Expression (GTEx) Project Version 8, incorporating both blood and kidney cortex tissues.

Genome-wide association study

Genotyping was performed using the UK BiLEVE Axiom array for 50,000 participants and the UK Biobank Axiom array for the remaining 450,000 participants. This yielded a total of 805,426 markers mapped to GRCh37 coordinates. GWAS analyses were conducted with plink2.0, excluding related individuals and fully adjusting for relevant covariates. The study included only unrelated White British participants, with adjustments for age, sex, and the first ten principal components to account for population stratification. Approximately 3% of participants were excluded due to failed genotype assays caused by insufficient DNA samples.

Mendelian randomization analysis

Mendelian randomization analyses were carried out using the TwoSampleMR R package (v0.6.4), with TREM2 gene expression as the exposure and obstructive nephropathy as the outcome. Instrumental variables (IVs) were selected based on genome-wide significance to minimize confounding by variants directly associated with the outcome rather than the exposure, and IVs with F statistics below 10 were excluded to avoid weak instruments. Multiple MR methods were applied, including

inverse variance weighted (IVW), MR-Egger, weighted median, simple mode, and weighted mode approaches. The primary analysis used IVW with a random-effects model, while MR-Egger analysis addressed potential horizontal pleiotropy. The weighted median method accommodated heterogeneity and outliers. Sensitivity analyses evaluated pleiotropy using the MR-Egger intercept P value and quantified heterogeneity with Cochran's Q statistic.

To examine the causal effect of TREM2 expression on obstructive nephropathy, common variants (minor allele frequency ≥ 0.05) associated with TREM2 expression in blood and kidney cortex tissues were selected from GTEx Project Version 8. Variants in high linkage disequilibrium (LD) were excluded. MR analyses accounted for LD among the selected variants.
